# Quasi-dark resonances with antiferromagnetic order in silicon metasurfaces

**DOI:** 10.1038/s41598-022-16167-6

**Published:** 2022-07-28

**Authors:** D. C. Zografopoulos, J. F. Algorri, J. M. López-Higuera, H. E. Hernandez-Figueroa, V. Dmitriev

**Affiliations:** 1 Consiglio Nazionale delle Ricerche, Istituto per la Microelettronica e Microsistemi, 00133 Rome, Italy; 2 Photonics Engineering Group, University of Cantabria, 39005 Santander, Spain; 3 CIBER-bbn, Instituto de Salud Carlos III, 28029 Madrid, Spain; 4 Instituto de Investigación Sanitaria Valdecilla (IDIVAL), 39011 Santander, Spain; 5 School of Electrical and Computer Engineering, University of Campinas-UNICAMP, Campinas, 13083-852 Brazil; 6 Electrical Engineering Department, Federal University of Para, Agencia UFPA, PO Box 8619, Belem, Para CEP 66075-900 Brazil

**Keywords:** Metamaterials, Nanophotonics and plasmonics

## Abstract

Quasi-dark resonances exhibiting antiferromagnetic order are theoretically investigated in a near-infrared metasurface composed of square slotted rings etched in a thin silicon layer on glass substrate. Access to the quasi-dark mode is achieved by reducing the symmetry of the metasurface according to the findings of a detailed group theory analysis. A thorough finite-element study reveals the key optical properties of the antiferromagnetic order quasi-dark mode, namely resonant wavelengths, quality factors, angular dispersion, and its robustness against optical extinction losses. It is demonstrated that the thickness of the silicon metasurface can adjust the asymmetry degree of the resonant Fano lineshape without affecting substantially its quality factor. Furthermore, tuning of the resonant wavelength can be achieved without significant modification of the Fano lineshape by controlling the angle of incidence of the impinging planewave. Overall, the work presents an all-dielectric, near-infrared metasurface for the excitation of sharp resonances with antiferromagnetic order, which can find use in emerging applications based on this particular configuration of artificial optical magnetism and/or strong field confinement and light-matter interaction.

## Introduction

Metamaterials, as well as metasurfaces, their two-dimensional analog, are artificial materials that have unusual electromagnetic properties, which stem from their designed structure rather than their composition. They are usually made of non-diffracting periodic arrays of unit cells, or “meta-atoms”, whose maximum dimension is smaller than the working wavelength. Among the various breakthroughs in electromagnetic wave manipulation enabled by metamaterials and metasurfaces^1–7^, their magnetic response at optical frequencies, known as artificial magnetism, stands out, as common natural materials have very weak magnetic response at infrared or visible frequencies. In contrast, metamaterials can be engineered to exhibit target magnetic properties not found in nature, such as negative permeability^8^.

In the case of metallic structures, this artificial magnetism is based on the inductive response of noble metals^9^. Some resulting applications have been the demonstration of left-handed-media^10,11^ and the propagation of magnetic plasmons over long distances with lower dissipation than electric plasmons^12,13^. Interestingly, a new plasmonic behaviour is demonstrated in Ref.^13^, a so-called antiferromagnetic (AFM) plasmon. Thanks to an antiphase plasmon propagation via the excitation of alternating ring currents due to strong near-field coupling, optical antiferromagnetism (OAFM) is demonstrated. The antiferromagnetic mode resembles antiferromagnetism in natural materials, being internally magnetic but resulting in zero net magnetic moments. Moreover, it is subradiant in nature and exhibits a field decay length of 2.65 ${\upmu }$m at the near-infrared (NIR) spectrum, which is seven times greater than a typical electric plasmon waveguide. The concept of antiferromagnetism has also been experimentally demonstrated at THz frequencies in metallic metasurfaces, the 2D variant of metamaterials, using split-ring resonators (SRR) with trapped modes and anti-parallel current configurations^14,15^.

One way to boost the optical response of metamaterials is to use dielectric materials, which can have very low to negligible losses in the visible and NIR spectrum. It has been established that electrically small nonmagnetic dielectric particles can exhibit a strong magnetic behaviour in Mie-like resonances due to the circulation of displacement currents, without contributing to Joule’s heating^16^. OAFM has been demonstrated in hybrid dielectric–plasmonic metasurfaces combining metallic SRR with dielectric spheres^17^. In such systems, the near field coupling can switch the ferromagnetic-like magnetization to antiferromagnetic at a certain distance. This concept was experimentally demonstrated in 2017 in the low-GHz range^18^. In the visible spectrum, OAFM has been demonstrated by using an all-dielectric metasurface of solid and hollow Si nanocylinders^19^.

More recently, OAFM has been studied in the context of quasi-dark or quasi-bound states in the continuum (BIC) resonances, in symmetry-broken, all-dielectric metasurfaces^20–24^, which combine the characteristic staggered magnetic dipole alignment resembling the antiferromagnetic order (AFMO) spin structure in natural antiferromagnetic materials with the very sharp spectral features and high-quality factors (*Q*-factors) associated with quasi-dark modes. It is remarked that this comparison serves as an analogy, as the magnetic dipole alignment mode in AFM materials is a static effect resulting from the electron spin, in contrast to the FMO/AFMO-like dynamic oscillation of optical magnetic dipoles in neighbouring metasurface cells. To experimentally excite and observe such resonances, the higher symmetry of a periodic metasurface array of dielectric resonators is perturbed resulting in symmetry reduction. This can be achieved by displacement of the resonator position, by locally changing the refractive index of selected resonators or by introducing eccentric holes in their shape, in order to open access to the quasi-dark, antiferromagnetic resonance^25,26^. Apart from demonstrating both FMO and AFMO of optically induced magnetic dipole moments, polarization conversion was also experimentally demonstrated in a low-symmetry, all-dielectric metasurface^26^.

Other recent proposals have been based on toroidal dipolar BIC, in an asymmetric metasurface formed by dielectric tetramer clusters of nanocylinders. In that study, the toroidal and antiferromagnetic effects of an asymmetric metasurface remain stable as the asymmetry degree increases and the polarization of the incident light changes^27^. As a general remark, although the AFMO can be relatively easily achieved in metallic or plasmonic metasurfaces^14,15^, it is not trivial to excite in purely dielectric systems working in the NIR^26^.

Interestingly, this novel phenomenon presents some intriguing characteristics both from the fundamental standpoint and in terms of potential applications. OAFM shows high-frequency dynamics and it allows for dense packaging of resonating elements, as adjacent particles do not magnetically interact. Moreover, the net magnetic moment is zero, which implies that stored information in the OAFM moment would be invisible to conventional magnetic probes and insensitive to disturbing magnetic fields. This aspect has raised considerable attention recently, and the possibilities to efficiently manipulate and detect the magnetic state of an antiferromagnet are reviewed in Ref.^28^. Another potential use of OAFM has been proposed in all-optical magnetic recording and the transport of electrical signals through dielectric media^17^. Furthermore, it has been shown that by controlling the parity of the magnetic dipoles in OAFM metastructures one can achieve light spin-selective light transmission, which can enable multiplexing of orthogonal circular polarization channels. These properties have been demonstrated in direction-controlled meta-holograms in the visible, based on a metasurface of hydrogenated amorphous silicon nanobricks^29^, and in the ultraviolet, by employing miniaturized half-waveplate nanobricks of Nb$_2$O$_5$ in a metasurface designed for ultraviolet vectorial anti-counterfeiting applications^30^. In addition, robust narrowband thermal emission has been recently demonstrated based on OAFM in a silicon metagrating in the NIR^31^.

In this work, we present a silicon metasurface supporting NIR-AFMO quasi-dark resonances, formed by a periodic array of square slotted rings (SSR), based on a recently proposed paradigm^32^. The initial $C_{4v}$ symmetry of the metasurface is reduced to $C_s$ by dislocating the centers of two SSR in a quadrumer supercell, thus opening access to the quasi-dark AFMO mode.

First, the symmetry properties of the metasurface and the polarization selection rules for the excitation of the target AFMO mode are studied by a group theory analysis. Subsequently, the key optical properties of the AFMO resonance, namely resonant wavelengths and *Q*-factors as a function of the degree of asymmetry, the effect of losses in the system, and angular dispersion, are thoroughly investigated by means of finite-element eigenvalue and full-wave simulations. The metasurface thickness is shown to minimally influence the *Q*-factor of the AFMO resonance, while it provides a direct means to adjust the asymmetry of its Fano lineshape. Typical absorption losses in crystalline silicon do not affect the obtainable *Q*-factors in realistic scenarios. Finally, tilting the metasurface and thus adjusting the angle of incidence provides a simple way to tune the AFMO resonant wavelength without significantly affecting its lineshape. Such induced magnetic ordering and strong resonances in all-dielectric NIR metasurfaces can find use in novel AFMO-based applications^26,28^.

## Metasurface structure and symmetry analysis

### Metasurface layout

**Figure 1:**
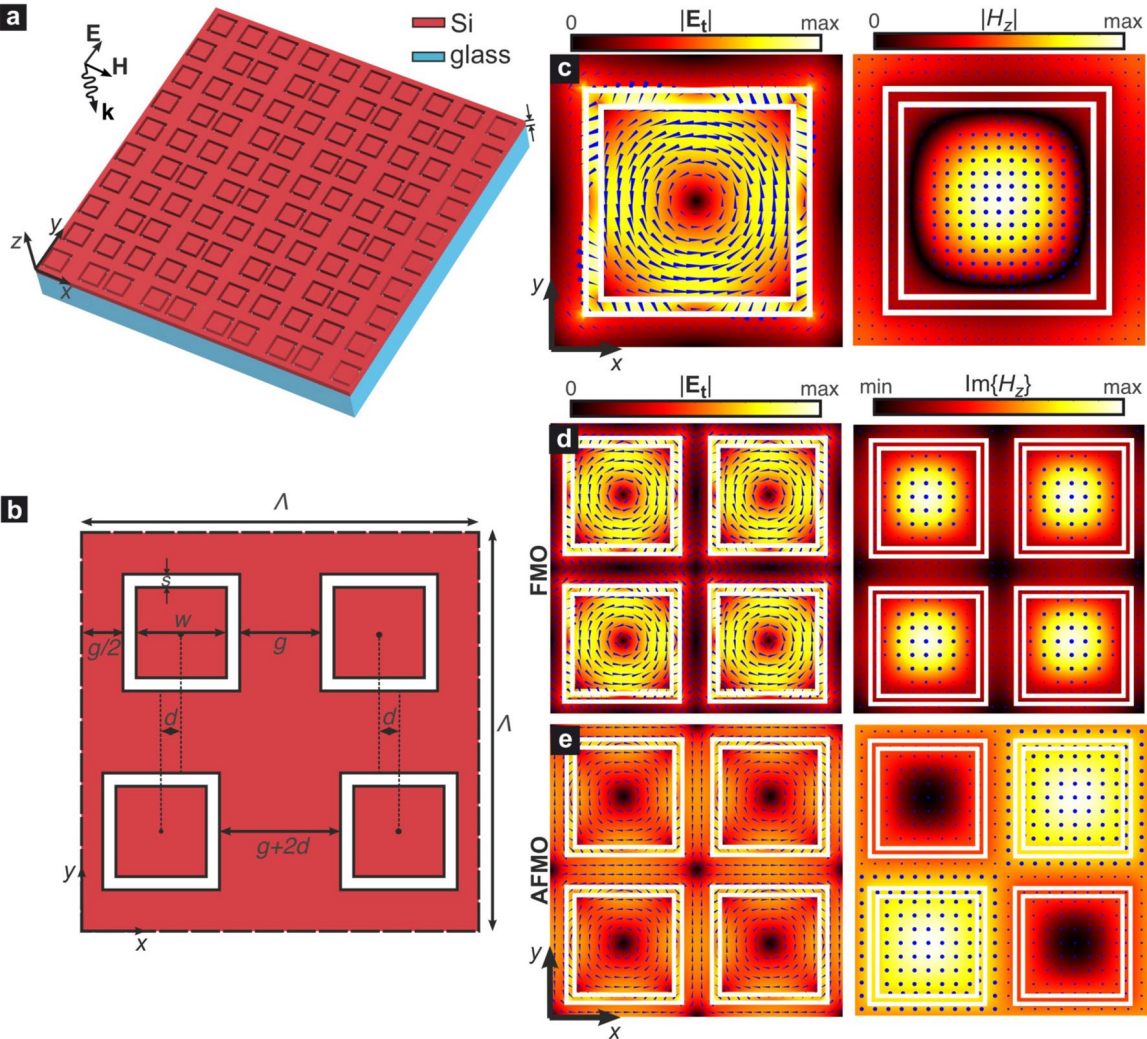
(**a**) Three dimensional layout of the dielectric metasurface of square slotted rings etched in a silicon layer of thickness *h*. (**b**) Cross-section of the metasurface unit cell composed of four rings, where the centers of the bottom row are laterally displaced towards the edge of the unit cell by a distance *d*. (**c**) Electric and magnetic field profiles of the circular dark mode supported by the symmetric metasurface, calculated by eigenvalue analysis ($\lambda _\text {res}^\text {CM}=1.2627$ ${\upmu }$m). (**d**) Field profiles of the symmetric supermode of the four-SSR metasurface unit cell, showing ferromagnetic order ($\lambda _\text {res}^\text {FMO}=\lambda _\text {res}^\text {CM}$). (**c**) Field profiles of the antisymmetric supermode of the four-SSR metasurface unit cell, showing antiferromagnetic order ($\lambda _\text {res}^\text {AFMO}=1.7311$ ${\upmu }$m).

The investigated all-dielectric metasurface is composed of a periodic pattern of SSR etched throughout a silicon layer of thickness *h*, as shown in Fig. 1a. The metasurfaces lies on an optically thick silica substrate. The SSR define silicon cuboids with $w \times w$ square cross-section and thickness *h*. The slot width is *s* and the interslot distance is *g*. In order to allow for the excitation of the AFMO quasi-dark mode, as it will be thoroughly explained in the next subsections, a supercell geometry is formed by $2\times 2$ SSR, where the centers of the two SSR in the bottom row are laterally displaced by a distance *d* towards opposite directions, as indicated in Fig. 1b.

In the general case of an asymmetric structure ($d \ne 0$) the pitch of the periodic array of quadrumer SSR equals $\Lambda =2(w+2s+g)$. In the non-perturbed case ($d=0$) the pitch reduces to $\Lambda /2$ and the metasurface shows $C_{4v}$ symmetry. In the following analysis, the geometry is defined by $w=330$ nm, $s=30$ nm, $g=110$ nm and hence $\Lambda =1$ ${\upmu }$m. Finally, the refractive index for silicon and silica in the calculations was $n_\text {Si}=3.48$ and $n_g=1.445$, respectively, and the silica glass substrate was modeled as a semi-infinite medium.

Regarding its practical demonstration, the metasurface can be fabricated following a standard electron beam lithography (EBL) process^33^. A polycrystalline silicon film is first deposited on the dielectric substrate by means of, e.g., low pressure chemical vapor deposition. Subsequently, the silicon film is patterned by EBL definition of its structure and reactive ion etching. It is remarked that the smallest feature in the proposed design is the slot width of 30 nm, which is fully compatible with the current resolution of EBL systems. As it will be thoroughly discussed, the critical parameter for the metasurface performance is *d*, which, however, refers to the displacement of the SSR center and not some geometrical feature. Hence, *d* can be much better controlled, as the spatial positioning of the raster system that moves the beam in EBL equipment can achieve sub-nm control^34^. In fact, the approach of symmetry-breaking by controlling the position of dielectric resonators and not by altering their shape has been shown to offer enhanced control over the achievable *Q*-factors in qBIC-resonant dielectric metasurfaces^22,35^.

### Ferromagnetic and antiferromagnetic dark supermodes

**Figure 2:**
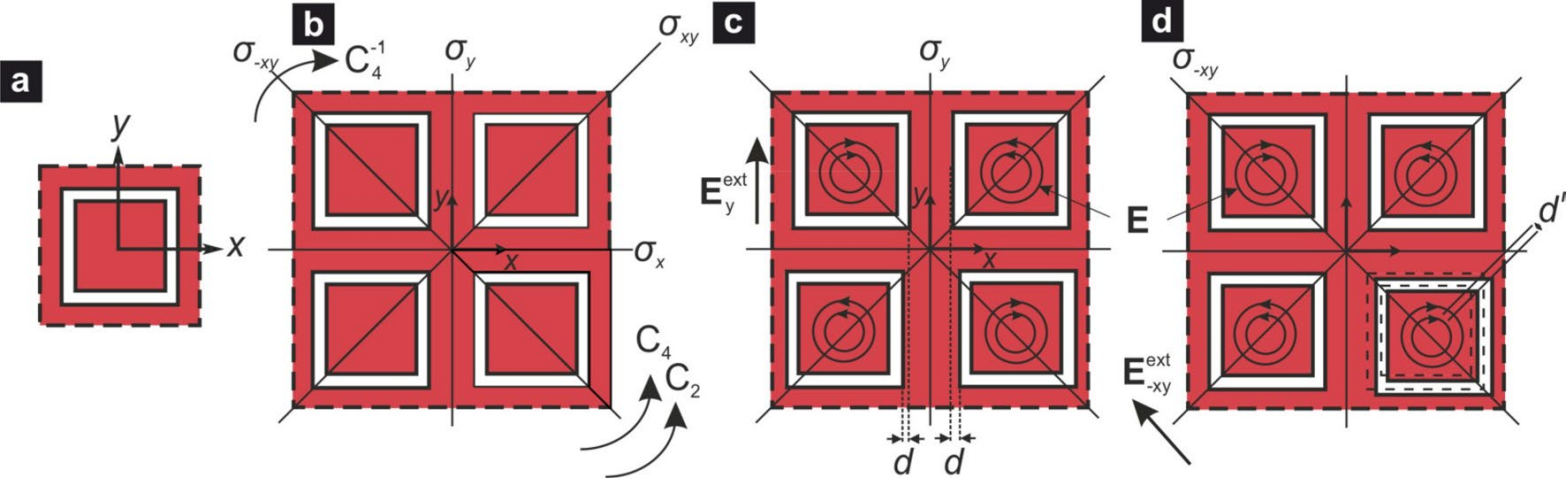
(**a**) Unit cell with $C_{4v}$ symmetry composed of a single SSR; (**b**) non-perturbed unit supercell, also with $C_{4v}$ symmetry, and its elements of geometrical symmetry; (**c**) perturbed unit supercell for AFMO mode excitation, symmetry $C_s^y$, *d* is the displacement of the unit cells along the *x*-direction; (**d**) perturbed unit supercell for AFMO mode excitation, symmetry $C_s^{-xy}$, $d'$ is displacement of the unit cell along the diagonal direction (the dashed squares depict the nonperturbed unit cell). In (**c**) and (**d**) $\mathbf{E}_y^{\text {ext}}$ and $\mathbf{E}_{-xy}^{\text {ext}}$ are the electric fields of the linearly polarized incident wave and $\mathbf{E}$ is a simplified depiction of the in-plane electric field of resonant mode in the unit cell.

We have recently shown that unperturbed SSR silicon metasurfaces support a series of modes who belong to different symmetry groups with respect to a linearly-polarized, normally-incident planewave, and hence they are dark, namely they cannot be excited under those circumstances^32^. Among these, there is one with a characteristic circular profile of the tangential electric field, which is reminiscent of the TE$_{01\delta }$ mode of a dielectric resonator^25^. The electric and magnetic field profiles of this mode at the mid-plane of the investigated metasurface ($z=h/2$, where $z=0$ is the plane of the interface between the metasurface and the glass substrate) are shown in Fig. 1c for $h=250$ nm, calculated by finite-element eigenfrequency simulations in Comsol Multiphysics. Floquet periodic boundary conditions (PBC) were applied at the lateral walls of the simulated unit cell with wavevector components $k_x=k_y=0$, i.e., at the $\Gamma$ point of the first Brillouin zone of the square lattice, since we are interested in the case of normal incidence. Perfectly matched layers (PML) were placed at sufficient distance from the metasurface along the *z*-axis in order to terminate the computational domain and provide accurate calculations of the complex eigenfrequencies of the structure. The PML were backed by scattering boundary conditions.

Figure 1c shows that the tangential electric field is confined mostly at the cuboid cross-section, with some field also concentrated in the square slot. The normal component $E_z$ is more than three orders of magnitude smaller than the tangential $\left| \mathbf {E_t} \right|$, verifying the planar circular profile of the mode’s electric field. The magnetic field is perpendicularly polarized with respect to the metasurface plane, with a field maximum at the cuboid center, thus leading to a net magnetic moment along the *z*-axis. The mode resonant wavelength of the considered circular mode (CM) is $\lambda _\text {res}^\text {CM}=1.26275$ ${\upmu }$m.

**Figure 3:**
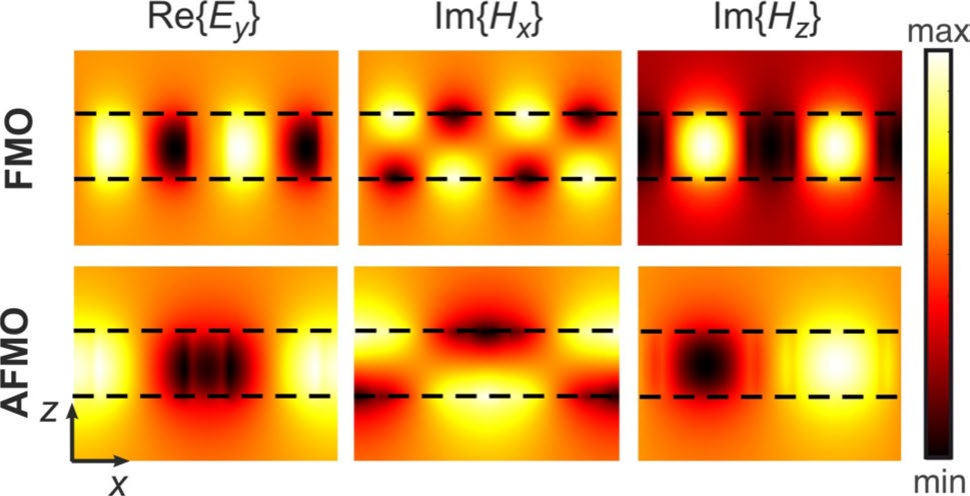
Profiles of the dominant field components for the FMO and AFMO supermodes of Fig. 2 on the $y=\Lambda /4$ plane. Due to the strong field confinement in the high-index silicon metasurface both supermodes are quasi-symmetric with respect to the $z=h/2$ plane with even electric field parity (even quasi-TE modes). The $H_z$ profile for the AFMO supermode shows the characteristic odd symmetry with respect to the $x=\Lambda /2$ axis.

Next, we perform a similar eigenfrequency analysis for the symmetric case ($d=0$) of the quadrumer supercell of Fig. 1b. In this case the unit cell is composed of four resonators, whose coupling leads to supermodes with symmetric or antisymmetric field profiles. The profiles of two such supermodes are shown in Fig. 1d,e. In the first case, the supermode is symmetric, with a periodic superposition of the profile of the isolated mode of Fig. 1c, and the same resonant wavelength. In the second case, however, the supermode shows an antisymmetric profile in a staggered configuration. The handedness of the electric field in-plane circular orientation is opposite in adjacent resonators, which leads to antiparallel out-of-plane magnetic field vectors, namely the characteristic AFMO configuration. The net magnetic moment over the entire supercell is zero, as adjacent magnetic moments cancel each other. The resonant wavelength of the AFMO supermode is $\lambda _\text {res}^\text {AFMO}=1.7311$ ${\upmu }$m, larger than $\lambda _\text {res}^\text {FMO}$, which is consistent with what expected for the antisymmetric supermode in coupled resonators.

It is interesting to note that although the main features in the field profiles of both FMO and AFMO supermodes are similar, the AFMO supermode shows a more delocalized field profile with the electric and magnetic fields distributed almost over the entire plane. This characteristic implies that the two modes will not have the same *Q*-factors when excited as quasi-dark resonances in symmetry-broken metasurfaces, as it will be discussed.

### Symmetry analysis

**Table 1:** IRREPs of group $C_{4v}$, mode order and transformation of 2D electric field $\mathbf{E}^{\text {ext}}$ of incident wave.

$C_{4v}$	e	$C_{2}$	$C_{4}$	$C^{-1}_{4}$	$\sigma _{x}$ $(y=0)$	$\sigma _{y}$ $(x=0)$	$\sigma _{xy}$	$\sigma _{-xy}$	Mode order,
									$\mathbf{E}^{\text {ext}}$-field
$A_1$	1	1	1	1	1	1	1	1	
$A_2$	1	1	1	1	$-1$	$-1$	$-1$	$-1$	Dark FMO
$B_1$	1	1	$-1$	$-1$	1	1	$-1$	$-1$	Dark AFMO
$B_2$	1	1	$-1$	$-1$	$-1$	$-1$	1	1	
*E*	$$\left( \!\!\! \begin{array} {cc} 1 &{} 0 \\ 0 &{} 1 \end{array} \!\! \right)$$	$$\left( \! \begin{array} {cc} \!\!\!-1 &{} 0 \\ 0 &{} \!\!\!\!-1 \end{array} \!\! \right)$$	$$\left( \!\!\! \begin{array} {cc} 0 &{}\!\!\!-1 \\ 1 &{} 0 \end{array} \!\! \right)$$	$$\left( \! \begin{array} {cc} 0 &{} 1 \\ \!\!\!-1 &{} 0 \end{array}\!\! \right)$$	$$\left( \!\! \begin{array} {cc} 1 &{} 0 \\ 0 &{} \!\!\!\!-1 \end{array}\!\! \right)$$	$$\left( \! \begin{array} {cc} \!\!\!-1 &{} 0 \\ 0 &{} 1 \end{array}\!\! \right)$$	$$\left( \! \begin{array} {cc} 0 &{} 1 \\ 1 &{} 0 \end{array}\!\! \right)$$	$$\left( \!\! \begin{array} {cc} 0 &{} \!\!\!-1 \\ \!\!-1 &{} 0 \end{array}\!\! \right)$$	$$\mathbf{E}^{\text {ext}}=\left( \!\begin{array} {c} E_x \\ E_y \end{array} \!\! \right)$$

**Table 2:** IRREPs of group $C_{2v}$, mode order.

$C^v_{2v}$	*e*	$C_2$	$\sigma _{x}$ $(y=0)$	$\sigma _{y}$ $(x=0)$	Mode order
$A_1$	1	1	1	1	Dark AFMO
$A_2$	1	1	$-1$	$-1$	Dark FMO
$B_1$	1	$-1$	1	$-1$	
$B_2$	1	$-1$	$-1$	1

**Table 3:** Symmetry degeneration table of group $C_{4v}$^36^.

$C_{4v}$	$C^v_{2v}$	$C^d_{2v}$	$C^v_{s}$	$C^d_{s}$
$A_1$	$A_1$	$A_1$	*A*	*A*
$A_2$	$A_2$	$A_2$	*B*	*B*
$B_1$	$A_1$	$A_2$	*A*	*B*
$B_2$	$A_2$	$A_1$	*B*	*A*
*E*	$B_1$, $B_2$	$B_1$, $B_2$	*A*, *B*	*A*, *B*

**Table 4:** IRREPs of group $C_s$, mode order and exciting $\mathbf{E}^{\text {ext}}$-field.

$C_{s}$	*e*	$\sigma _{y} (x=0)$	Mode order (exciting field)
*A*	1	1	Bright AFMO ($E_y$)
*B*	1	$-1$	Bright FMO ($E_x$)

**Table 5:** IRREPs of group $C_d$, mode order and exciting $\mathbf{E}^{\text {ext}}$-field.

$C_{d}$	*e*	$\sigma _{-xy}$	Mode order (exciting field)
*A*	1	1	
*B*	1	$-1$	Bright FMO, bright AFMO ($E_{-xy}$)

Here we present a symmetry analysis of the investigated metasurface, in order to identify the conditions under which the target AFMO can be excited by a normally impinging, linearly polarized planewave with electric field $\mathbf{E}^{\text {ext}}$. The 2D (in the *x*–*y* plane) group of geometrical symmetry of the unit cell of the non-perturbed metasurface shown in Fig. 2a is $C_{4v}$ in Schoenflies notation^37^. As discussed, the electric field of the desired eigenmode inside the $w \times w$ dielectric square cross-section of the unit cell assumes an almost circular profile, shown in Fig. 1c. Therefore, to simplify the symmetry analysis we shall use in the following discussion the geometry of electric field in the unit cell presented by concentric circles.

In order to excite the dynamical AFMO resonance we need four unit cells combined in the supercell shown in Fig. 2b. The symmetry of the non-perturbed supercell is also $C_{4v}$. In Table 1, the irreducible representations (IRREPs) of the group $C_{4v}$ are presented and the transformations of the electric field $\mathbf{E}^{\text {ext}}$ in the last column by different symmetry operators are defined by the 2D-IRREP *E*. The analysis of this Table demonstrates that in symmetry $C_{4v}$ of the supercell both the FMO and AFMO resonances are inaccessible, since the FMO (AFMO) electric field profile belongs to IRREP $A_2$ ($B_1$), but the electric field of incident wave $\mathbf{E}^{\text {ext}}$ does not have the symmetry $C_{4v}$.

**Figure 4:**
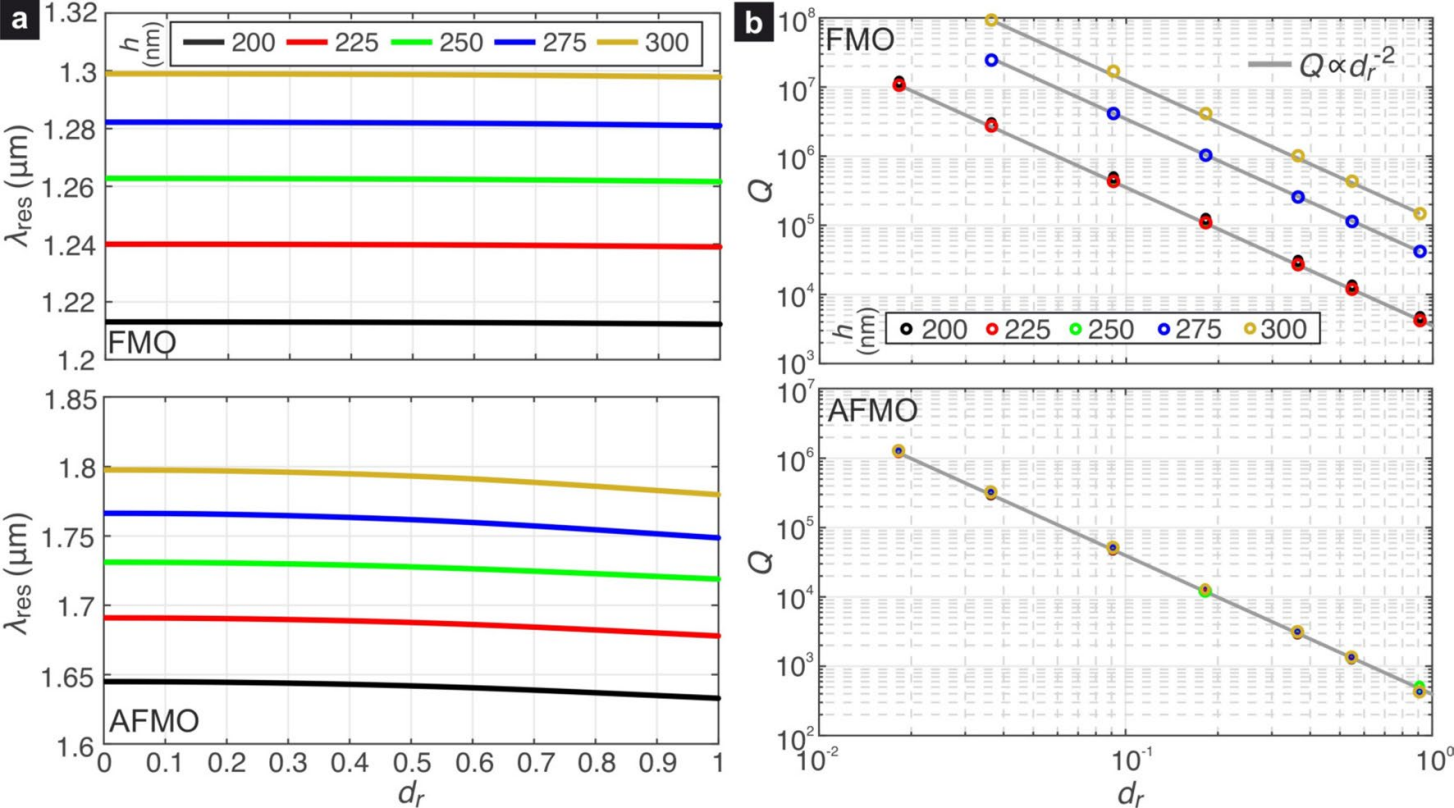
(**a**) Resonant wavelengths of the FMO/AFMO quasi-dark modes as a function of the relative displacement $d_r$ and for various values of the Si layer thickness *h*. (**b**) Quality factors of the FMO/AFMO for the same set of parameters as in (**a**). The *Q* values follow the $Q\propto d_r^{-2}$ law, characteristic of quasi-dark resonances in symmetry-broken metasurfaces.

In order to open access for the target AFMO resonance, one should lower the symmetry of the supercell. A reduction of symmetry $C_{4v}$ to $C_{2v}$ does not produce the desired effect. This can be seen in Table 2, showing the IRREPs of the group $C_{2v}$, and in Table  3 where the symmetry degeneration scheme of the group $C_{4v}$ to lower ones is presented.

Nevertheless, a reduction of symmetry $C_{4v}$ to $C_{s}$ or $C_{d}$, whose IRREPs are given in Tables 4 and 5, respectively, can provide such access because there are corresponding components of $\mathbf{E}^{\text {ext}}$ (see Fig. 9 of^36^). It is interesting to note that in the symmetry $C_{s}$ the FMO and AFMO supermodes can be excited by orthogonal components of the external field $E_x$ and $E_y$, respectively, but in the case $C_{d}$, both the FMO and AFMO modes can be excited by the same diagonal linear polarization of the field $E_{-xy}$ .

The reduction of symmetry can be fulfilled by many ways. Here, we focus on the case shown in Fig. 1b, or equivalently Fig. 2c, which involves the displacement of the centers of two SSR in the supercell. More generally, for instance in the case of $C_{d}$, one can change the resonant frequency of one of the unit cells by adjusting its dimensions (such as *w* and *s*), by changing the geometry of the unit cell from square to rhombic one, or by displacing one of the unit cells along the diagonal by distance $d'$, as in Fig. 2d.

Now we shall apply to the 3D symmetry of the metasurface. With the presence of the horizontal middle plane of symmetry $\sigma _h$, i.e. $z=h/2$, the discussed resonance can be classified as a TE mode. Strictly speaking, in the considered structure there is no such plane because the refractive indices of silica (the glass substrate with $n_g=1.445$) and air (above the structure, $n=1$) are different. However, with such a relatively small difference between the indices and given their large index contrast with silicon, one can consider an approximate symmetry with element $\sigma _h$ and the corresponding modes as quasi-TE. Figure 3 shows the profiles of the major field components for the FMO and AFMO supermodes studied in Fig. 1d,e, calculated at the *x*–*z* plane $y=\Lambda /4$. As verified also in Fig. 1d,e, the electric field is *y*-polarized on that plane. The field profiles of both supermodes are quasi-symmetric with respect to the $\sigma _h$ plane, owing to the strong field confinement in the metasurface layer. The parity of the electric field in both cases is even, so both supermodes are classified as quasi-TE even modes. The FMO shows the expected periodic pattern in all field components, as the supercell contains four identical resonators. On the contrary, the $H_z$ component of the AFMO supermode shows the characteristic odd symmetry with respect to the $x=\Lambda /2$ plane, namely the characteristic staggered magnetic field configuration of the AFMO.

## Results

### Resonant wavelengths and *Q*-factors

Here we calculate by means of an eigenfrequency analysis the resonant wavelengths and the *Q*-factors of the quasi-dark FMO and AFMO resonant modes in the perturbed metasurface of Fig. 1b. We define an asymmetry parameter $d_r=d/d_m$, where $d_m$ is the maximum displacement equal to $d_m=g/2=55$ nm. We parametrize the analysis in terms of *h*, which varies between 200 and 300 nm, namely a typical thickness range in silicon-on-glass or silicon-on-insulator wafers.

Figure 4a shows the resonant wavelengths of the two supermodes as a function of $\{d_r,h\}$. The increase of the silicon thickness leads to higher $\lambda _\text {res}$ as more high-index material is introduced in the metasurface. For small values of $d_r$, the $\lambda _\text {res}$ remain almost invariant, although as $d_r$ tends towards $d_m$ and the metasurface geometry is modified a reduction of $\lambda _\text {res}$ is observed, particularly in the case of the AFMO supermode.

The corresponding *Q*-factors for both resonant supermodes are reported in Fig. 4b. As expected, larger degrees of asymmetry lead to a rapid reduction of the *Q*-factor. The dependence $Q(d_r)$ follows the inverse square law $Q\propto d_r^{-2}$, which is characteristic of quasi-dark resonances in symmetry-perturbed metasurfaces in the non-diffractive regime^19^. Furthermore, it is noticed that for a given degree of asymmetry $d_r$ the FMO supermode provides always larger by at least one order of magnitude *Q*-factors compared to the AFMO supermode. For higher thicknesses of the silicon layer the FMO resonance manifests larger *Q*-factors, whereas the *Q* of the AFMO resonance depends marginally on *h* for the considered range.

The higher *Q*-factors of the FMO compared to the AFMO resonance for a given degree of asymmetry can be attributed to the electric field profiles of the two resonant modes, shown in Fig. 1d,e, where it is shown that the field of the FMO mode has an antiparallel orientation in the region between the square slots, namely where the symmetry perturbation is introduced. This leads to almost total cancellation of the in-plane electric field, leading to lower coupling with the external electric field, in contrast to the case of the AFMO mode, which demonstrates a much more uniform alignment of the electric field in the discussed region.

In addition, it is also shown in Figs. 1 and 3 that the FMO mode exhibits stronger confinement in the silicon cuboids defined by the square slots, whereas the AFMO mode is more delocalized. Such properties can be exploited, respectively, in applications like non-linear higher harmonic generation and sensing, where strong light-matter interaction with the environment is needed. We also remark that the two modes operate at different wavelengths and under orthogonal polarizations of the excitation wave, as demonstrated in the symmetry analysis. Therefore, the metasurface could operate in dual mode, probing two wavelengths and polarizations, while exploiting the particular field profiles of both the FMO and AFMO modes.

### Transmittance spectra

**Figure 5:**
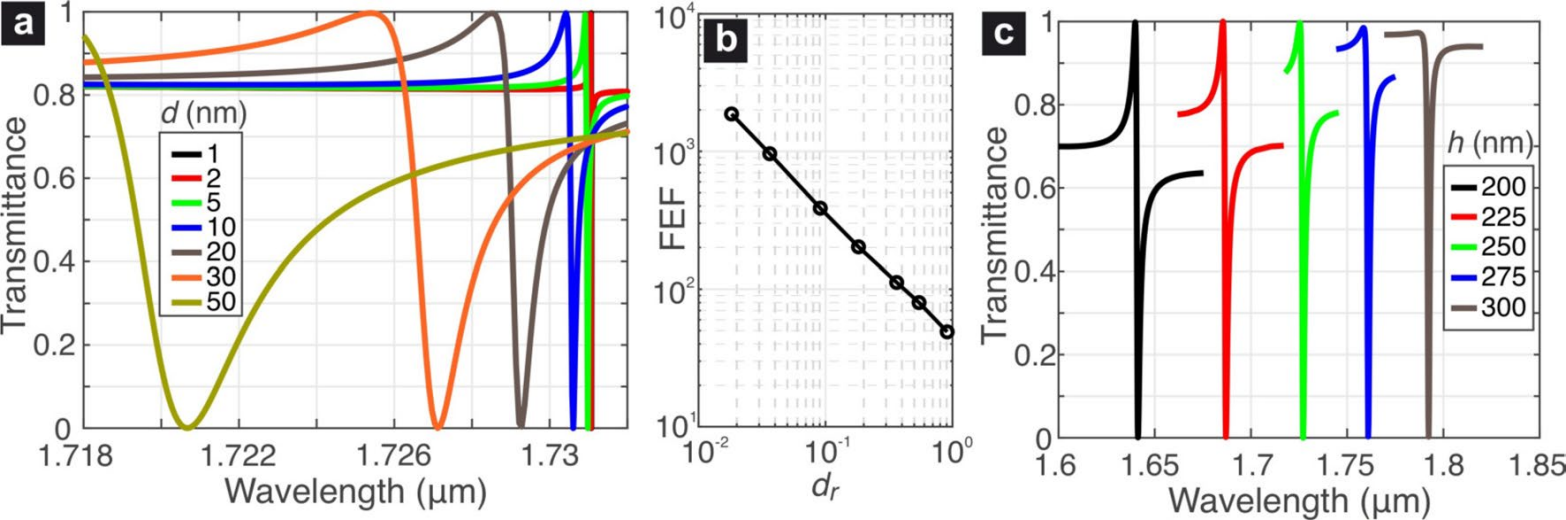
(**a**) Transmittance spectra in the vicinity of the AFMO quasi-dark resonance for $h=250$ nm and various values of *d* and (**b**) corresponding local field-enhancement factors. (**c**) Transmittance spectra in the vicinity of the AFMO quasi-dark resonance for $d=30$ nm and various values of *h*. Adjusting the Si thickness *h* allows for tuning the asymmetry of the Fano AFMO resonance without noticeably affecting its *Q* factor.

Next we focus on the target AFMO supermode and we calculate the transmittance spectra for an indicative set of geometries by means of full-wave finite-element simulations. The impinging planewave is *y*-polarized, in accordance with the polarization selection rules of Table 4 (an *x*-polarized planewave cannot excite the AFMO resonance). First, we consider the case where $h=250$ nm and we let the displacement value vary between $d=1$ and 50 nm, which covers almost entirely the range $d_r \in \left( 0,1 \right)$. The results are presented in Fig. 5a. The resonant spectra show an asymmetric Fano profile and their $\lambda _\text {res}$ decreases for higher *d* in accordance with the green curve in Fig. 4a. In addition, the resonance linewidth broadens, leading to lower *Q*-factors, as in Fig. 4b.

High *Q*-factors are usually associated with strong local field confinement, which can be quantified by means of the field enhancement factor (FEF) defined as: $\text {FEF}=\left| {\mathbf {E}} \right| / E_0$, where $E_0$ is the amplitude of the impinging planewave. We have calculated the electric field profile at the metasurface mid-plane and at the resonant wavelengths of the AFMO mode for the cases studied in Fig. 5a. The field profiles resemble the reference profile shown in Fig. 1e for the symmetric case ($d=0$), deviations being more noticeable for increasing values of *d*. The maximum FEF, identified among the electromagnetic hot spots of the resonant field spatial profiles, as a function of *d* is presented in Fig. 5b, exhibiting a trend similar to the associated *Q*-factors of Fig. 4b.

Figure 5c investigates the influence of the thickness *h* on the Fano profile of the AFMO resonances for a fixed displacement value $d=30$ nm corresponding to quality factors $Q\simeq 1300$. Apart from the expected red-shift in $\lambda _\text {res}$ for higher thickness, it is also verified that the *Q*-factor does not significantly depend on the value of *h*. More interestingly, the Fano profile of the resonance lineshape is affected by *h*. This provides a means of adjusting its profile, which can be useful from the application point of view, as the required Fano lineshape can be application-dependent^38–40^.

**Figure 6:**
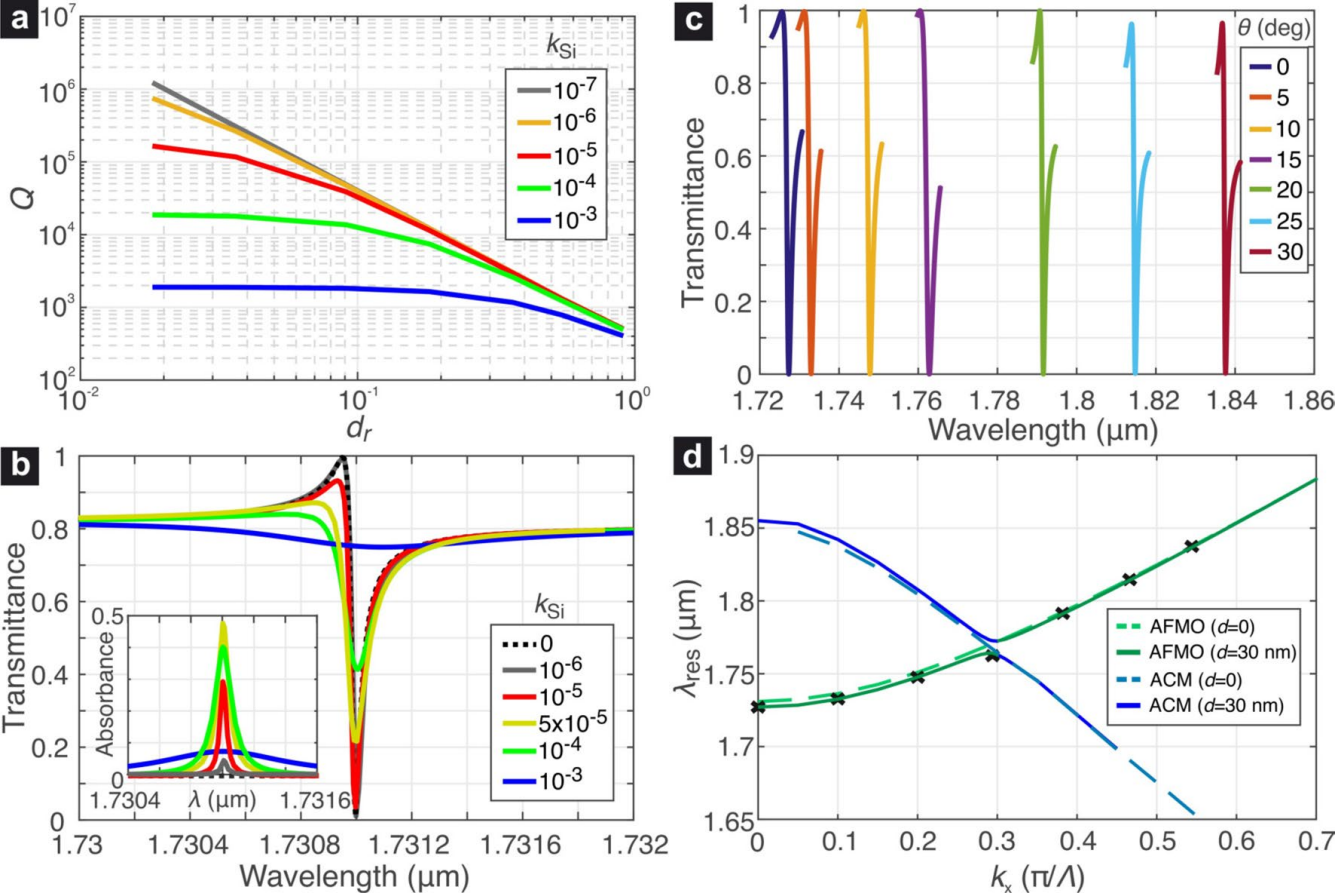
(**a**) Dependence of the *Q*-factor of the AFMO resonance on $k_\text {Si}$. (**b**) Transmittance and absorbance spectra of the AFMO resonance for $d=5$ nm and various values of $k_\text {Si}$. The spectrum for the lossless case is included as reference. (**c**) Transmittance spectra of the AFMO resonance calculated for oblique incidence with an angle $\theta =0$ to $30^{\circ }$ in steps of $5^{\circ }$ in the *x*–*z* plane and TE-polarized incident planewave. (**d**) Resonant wavelengths calculated by eigenvalue analysis for the AFMO resonance and an interfering mode for the symmetric ($d=0$) and an asymmetric metasurface ($d=30$ nm), showing avoided mode crossing in the perturbed case.

To provide more insight on this aspect, the calculated transmittance spectra were fitted to the Fano function

1 $$\begin{aligned} T(\omega _n) = C \dfrac{\left( F\gamma _n + \omega _n - 1\right) ^2}{\left( \omega _n-1\right) ^2+\gamma _n^2}, \end{aligned}$$

where *C* is a scaling parameter, *F* is the Fano parameter that described the degree of asymmetry, $\omega _n = \omega /\omega _0$ the circular frequency normalized to the resonant frequency $\omega _0$, and $\gamma _n$ is the normalized Fano linewidth. The parameter of interest in this analysis is the asymmetry factor *F*. For a value $F=0$, the Fano profile corresponds to a symmetrical dip with zero transmittance at $\lambda _\text {res}$, i.e., a standard Lorentzian lineshape. When $F=1$ the Fano profile is asymmetric with $\lambda _\text {res}$ at half the distance between the minimum and maximum transmittance wavelengths^38^.

For the set of spectra shown in Fig. 5c, the corresponding values of *F*, as calculated through the fitting procedure are $h:\{200,225,250,275,300\}$ nm is $F:\{0.705,0.739,0.476.0.295.0.132\}$. By inspecting the profiles of the spectra, it is observed that for $h=300$ nm the resonance is almost Lorentzian ($F=0.132$), while for $h=225$ nm the lineshape is close to the characteristic Fano asymmetric profile ($F=0.739$). Therefore, it is deduced that the selection of the metasurface thickness provides a means to control the resonant lineshape, without affecting significantly its *Q*-factor.

### Impact of silicon losses

In this subsection, we provide an assessment of the impact of scattering, absorption and other material- or fabrication-induced loss mechanisms, which are quantified in this analysis by adding a loss coefficient *k*, i.e., imaginary part, to the refractive index of silicon as $n_\text {Si}=3.48-jk$. The impact of *k* on the achievable *Q*-factors for the AFMO resonance is evaluated first by an eigenfrequency analysis, whose results are shown in Fig. 6a. Due to stronger field confinement in silicon, the effect of losses is more profound for small asymmetry values $d_r$, tending to saturate the maximum obtainable *Q*-factor. It is remarked that the loss coefficients here considered are larger that intrinsic absorption losses in crystalline silicon^41^.

Figure 6b reports the transmittance spectra calculated for $d=5$ nm (namely $d_r=0.091$) and various *k* values, where $h=250$ nm. For a loss coefficient up to $k=10^{-5}$ the transmittance spectrum is marginally affected, as confirmed in the corresponding *Q*-factor values in Fig. 6a. Further increasing the losses progressively dampens the resonance, which becomes hardly distinguishable for $k=10^{-3}$ due to the reduced *Q*-factor and small modulation depth of the transmitted power.

The inset in Fig. 6b shows the corresponding absorbance spectra, which in the case investigated, namely a single resonator coupled with one input and one output radiation channels, is limited to a theoretical maximum of $50\%$^42^. This is achieved at the critical coupling condition, i.e., when the radiative and non-radiative decay rates are equal. Here, it occurs for a silicon extinction coefficient approximately $5 \times 10^{-5}$, as demonstrated by the respective absorbance spectrum, which almost reaches $50\%$ at the resonant wavelength. Given that the metasurface is non-diffracting, the reflectance can be calculated simply as $R=1-T-A$, where *R*, *T*, *A* are the reflectance, transmittance, and absorbance, respectively.

### Angular dispersion

Finally, we evaluate the angular dispersion of the AFMO supermode, namely the dependence of its resonant wavelength as a function of the angle of incidence. Under oblique incidence, the tangential component of the impinging planewave’s wavevector is non-zero (the qBIC resonances are excited at off-$\Gamma$ points), which changes the coupling conditions and can lead to significant angular dispersion, unless the structure is otherwise optimized^36^. Here, we consider a planewave obliquely incident in the *x*–*z* plane with an angle $\theta$ with respect to the *z*-axis. The planewave is TE-polarized, namely along the *y*-axis. We consider the indicative case for $d=30$ nm and first calculate the transmittance spectra of the AFMO resonance for $\theta =0$ to $30^{\circ }$ in steps of $5^{\circ }$. The results are shown in Fig. 6c, calculated in a spectral window of 6 nm centered at the resonant wavelength in each case. The selected spectra show similar Fano lineshapes with approximately the same degree of symmetry. Their *Q*-factors do not vary by a factor more than 2, in particular, $Q(0^{\circ })=1307$ vs. $Q(30^{\circ })=2443$. Hence, controlling dynamically the angle of incidence provides a simple but effective means of tuning the AFMO resonant wavelength.

To provide more insight on the dependence of $\lambda _\text {res}^\text {AFMO}(\theta )$, we calculate the resonant wavelengths for $d=0$ and 30 nm by means of an eigenvalue study, by varying the $k_x$ component of the wavevector applied at the Floquet PBC. In the first case the metasurface retains its $C_{4v}$ symmetry and, thus, the AFMO is not accessible neither under normal nor oblique incidence. Figure 6d shows in green dashed line the evolution $\lambda _\text {res}^\text {AFMO}(\theta )$ for $d=0$. It is interesting to note that its dispersion curve crosses that of another mode, which will be called further as avoided crossing (AC) mode, at $k_x^\text {AC} \simeq 0.3 \pi /\Lambda$. Since the AFMO mode is dark, the two modes do not interfere anyhow.

However, in the perturbed metasurface of $d=30$ nm the interference of these two modes results in AC and their dispersion curves split in an upper and lower branch^43^. Away from the critical point of $k_x^{\text {AC}}$ the resonance on the green continuous line is AFMO, while in its vicinity the AFMO mode hybridizes with the avoided crossing mode (ACM). The cross symbols in Fig. 6d are the $\lambda _\text {res}^\text {AFMO}(\theta _i)$ calculated by means of fitting the spectra of Fig. 6c with the Fano function of Eq. (1), showing perfect agreement. It is noticed that the spectrum for $\theta =15^{\circ }$, which corresponds to $k_x^{15^{\circ }}=0.294 \pi /\Lambda$ via the relation $k_x=\left( 2\pi /\lambda _\text {res}\right) \sin \theta$, namely very close to the AC point, exhibits a similar Fano lineshape compared to the rest of the calculated spectra, albeit with a somewhat lower $Q=909$.

## Conclusions

To summarize, we have theoretically demonstrated the excitation of quasi-dark resonances with AFMO in a silicon NIR metasurface with a simple slotted geometry, which can be readily fabricated with standard EBL equipment. The symmetry properties of the structure are thoroughly analyzed by means of group theory and the various possibilities for the excitation of the AFMO quasi-dark resonance in the investigated metasurface are discussed. Furthermore, we provide an analysis of the optical properties of the AFMO mode, which are important both from a fundamental and application point of view: resonant wavelengths, *Q*-factors, and Fano lineshapes as a function of the induced asymmetry and metasurface thickness, angular dispersion, and quenching effects of losses on the obtainable *Q*-factors. The characteristic staggered configuration of anti-parallel magnetic moments perpendicular to the metasurface can find use in flat-optics applications inspired by the physics of antiferromagnetic materials.

## Data Availability

The datasets generated during and/or analysed during the current study are available from the corresponding author on reasonable request.
